# ISRII 2025: Advancing equity in digital interventions across the lifespan - an introduction to this year's conference in San Diego, CA — August 4–7, 2025

**DOI:** 10.1016/j.invent.2025.100836

**Published:** 2025-05-23

**Authors:** Anna-Carlotta Zarski

**Affiliations:** Philipps-University Marburg, Division of eHealth in Clinical Psychology, Schulstraße 12, D-35032 Marburg, Germany

The International Society for Research on Internet Interventions (ISRII) is a global network dedicated to advancing the development, evaluation, and implementation of digital tools and therapeutics for mental and behavioral health. Founded in 2004 by researchers recognizing the growing potential of internet-based health applications, ISRII fosters collaboration, knowledge exchange, and innovation in digital health research.

ISRII promotes rigorous, user-centered research on a wide range of digital technologies — including apps, games, virtual reality, remote sensing, and robotics — designed to promote health, prevent illness, support treatment, and enhance long-term wellbeing. Central to ISRII's mission is understanding how individuals engage with digital interventions and how these tools can best support diverse needs, preferences, and care pathways.

Beyond advancing science, ISRII actively supports the implementation of digital health solutions across healthcare settings worldwide, with a particular focus on improving health equity and access to evidence-based care. ISRII unites researchers, clinicians, developers, policymakers, and industry partners globally, fostering inclusive collaboration and aiming to create effective, ethical, and scalable healthcare solutions that reach people everywhere — especially in underserved communities.

ISRII offers its members access to a vibrant, supportive, and globally connected community dedicated to advancing digital health research. Special Interest Groups provide opportunities for focused exchange on key topics such as implementation, diversity, and career development for early and mid-career researchers. Members can engage in exclusive events like international eHealth implementation talks, the eHealth Unpacked series for project insights, or networking opportunities through the ISRII Connection and Mentoring Program. Members are encouraged to actively shape the organization by joining the Board or taking on officer roles. ISRII also supports the visibility of its members' research by offering reduced Open Access publication fees for the journal Internet Interventions. The highlight of the ISRII community is its scientific conference, where members benefit from discounted registration and the chance to connect and collaborate with colleagues from around the world.

The 13th ISRII Conference will take place from August 4–7, 2025, at the University of San Diego in California. Under the theme *Advancing Equity in Digital Interventions Across the Lifespan*, the conference will bring together leading researchers, practitioners, and innovators to share cutting-edge research and explore the future of digital health. Conference Chairs Professor Stephen Schueller and Professor Adrian Aguilera warmly invite you to this three-day event filled with inspiring talks and discussions – plus a workshop day – all set in a collegial and welcoming atmosphere on the beautiful University of San Diego campus.

Highlights of ISRII 2025 include:

• Keynotes: A keynote panel on *Supporting Youth through Digital Interventions* will feature leading voices in research and practice. Bethany Teachman, Ph.D., Professor of Psychology at the University of Virginia, will present on scaling evidence-based online tools to promote mental health globally and fostering youth wellbeing in digital environments. Jana Haritatos, Ph.D., Chief Science Officer at Hopelab, will share insights on driving innovation at the intersection of science and technology to improve mental health outcomes for young people. David Ball, MBA, Head of Well-Being at SecondMuse, will discuss addressing global gaps in youth mental health support. Another keynote will highlight trailblazers in digital health research.
• Workshops: On Monday, August 4, 2025, prior to the conference, four practical half-day workshops will be offered, covering topics such as human-centered design in research, AI and machine learning for mental health, designing AI chatbots for digital interventions, and creating interactive digital programs using the Computerized Intervention Authoring System (version 3.0) without coding.
• Early- and Mid-Career Researcher (EMCR) Activities: Dedicated events will provide EMCRs with opportunities to network, connect with senior researchers, and build lasting collaborations — while having fun along the way.
• Awards: ISRII will honor outstanding contributions to digital mental health research, practice, and community engagement through its awards ceremony. Nominations can be submitted via isrii.org.
• Social Events: Several informal social events will create a collegial and memorable conference experience, including a festive conference dinner featuring an authentic Mexican buffet and plenty of opportunities to connect and celebrate ISRII's diverse community.
• Accommodation: Affordable university housing will be available on the University of San Diego campus for conference attendees.
• Special Issue in Internet Interventions: A special issue on “*Advancing Equity in Digital Interventions Across the Lifespan*” will highlight key research from the conference. Participants will be invited to submit their research following the conference.

For more information, the latest program updates, and to register, please visit https://isrii.org/isrii-13th-scientific-meeting-2025/. If you have any questions, feel free to contact info@isrii.org.

We look forward to welcoming you to ISRII13!

Anna-Carlotta Zarski, Emily Lattie, Stephen Schueller, and Adrian Aguilera on behalf of the ISRII13 Conference Committee

## Declaration of competing interest

The authors declare no known competing financial interests or personal relationships that could have appeared to influence the content of this article.

